# Impaired Consciousness, Acute Respiratory Distress Syndrome (ARDS), and Multiorgan Dysfunction Syndrome (MODS) As the Triad of Mortality in Severe Dengue: Evidence From 100 Consecutive ICU Admissions

**DOI:** 10.7759/cureus.113041

**Published:** 2026-07-20

**Authors:** Akanksha Gupta, Sunita Gupta, Divyam Goyal, Neel S Dave

**Affiliations:** 1 General Medicine, Maharishi Markandeshwar Institute of Medical Science and Research, Mullana, IND

**Keywords:** ards (acute respiratory distress syndrome), glassgow coma scale, icu mortality, impaired consciousness, multiorgan dysfunction syndrome, organ dysfunction triad, severe dengue

## Abstract

Background

Severe dengue is a leading contributor to intensive care unit (ICU) admissions and death in tropical regions. Identifying factors associated with poor outcomes early is critical for guiding treatment and allocating resources in critically ill patients.

Methods

This prospective observational cohort enrolled 100 consecutive patients with WHO 2009-defined severe dengue admitted to the ICU and Emergency Department of Maharishi Markandeshwar Institute of Medical Science and Research (MMIMSR), Mullana, Haryana, between March 2025 and January 2026. Clinical, laboratory, and severity-score data (Acute Physiology and Chronic Health Evaluation II (APACHE II), Sequential Organ Failure Assessment (SOFA)) were recorded and compared between survivors and non-survivors using appropriate statistical tests, relative risks (RR) with 95% CI, and ROC analysis.

Results

The mean age was 47.2 ± 17.5 years; 58% were male. ICU mortality was 21.0% (21/100). Thrombocytopenia showed no mortality link (p = 0.678). Higher pulse rate, respiratory rate, body temperature, total leucocyte count, creatinine, bilirubin, and lactate, together with lower blood pressure, Glasgow Coma scale (GCS) score, arterial pH, and PaO2, were each associated with ICU mortality (all p < 0.05). Among warning signs, only hepatomegaly >2 cm was significant (50.0% vs. 17.0% mortality; RR 2.93, 95% CI 1.41-6.09; p = 0.024). Impaired consciousness (72.4% vs. 0% mortality; RR 103.20, 95% CI 6.46-1649.18; p < 0.001), acute respiratory distress syndrome (ARDS; 100% vs. 12.2%; RR 7.55, 95% CI 4.33-13.16; p < 0.001), and multiorgan dysfunction syndrome (MODS; 76.9% vs. 12.6%; RR 6.08, 95% CI 3.25-11.39; p = 0.002) showed the strongest associations (φ = 0.807, 0.647, 0.531 respectively). APACHE II (AUC 0.935) and SOFA (AUC 0.938) had the best discriminative accuracy, followed by lactate (AUC 0.835) and arterial pH (AUC 0.824).

Conclusions

Impaired consciousness, ARDS, and MODS strongly predicted mortality in this severe dengue cohort, forming a clinically vital cluster for early risk stratification. These single-center findings question the thrombocytopenia-centric paradigm and support closer attention to neurological status and organ dysfunction from ICU admission onward.

## Introduction

Dengue virus infection, transmitted by *Aedes aegypti*, is a major mosquito-borne viral illness and an important public health challenge in tropical and subtropical regions. While most infections are self-limiting, a subset of patients develop severe dengue, which is associated with significant morbidity and mortality. The burden of severe dengue continues to increase in endemic countries, including India [1].

According to the WHO 2009 classification, severe dengue is characterized by severe plasma leakage, severe bleeding, and/or severe organ involvement. Complications such as dengue shock syndrome, acute respiratory distress syndrome (ARDS), acute liver injury, acute kidney injury (AKI), encephalitis, and multiorgan dysfunction syndrome (MODS) are associated with poor clinical outcomes and increased mortality [2].

Despite advances in supportive and critical care management, mortality remains substantial among patients with severe dengue, particularly those requiring intensive care. Early identification of patients at risk of poor outcomes is crucial for timely intervention and optimal resource utilization. Clinical features, laboratory abnormalities, and severity assessment scores such as Acute Physiology and Chronic Health Evaluation II (APACHE II) and Sequential Organ Failure Assessment (SOFA) have been evaluated as potential factors associated with mortality; however, findings have varied across studies and populations [3-6].

Periodic regional studies are necessary to better understand the clinical profile, factors associated with severity, and outcomes of dengue, as these may vary across populations. Although several studies have evaluated dengue infection with emphasis on thrombocytopenia and prognostic scoring systems, limited studies have been conducted in North India regarding severe dengue specifically aimed at identifying factors associated with outcome. Platelet counts drive transfusion decisions in most hospitals across South and Southeast Asia, and thrombocytopenia dominates the dengue severity narrative in clinical education.

Therefore, the present study was undertaken at a tertiary care teaching hospital to evaluate the clinical profile and outcomes of patients with severe dengue and to identify factors associated with mortality. Such information may aid in early risk stratification and improve the management of critically ill dengue patients.

## Materials and methods

Study design and setting

This was a prospective, hospital-based, observational cohort study conducted at the ICU and Emergency Department of Maharishi Markandeshwar Institute of Medical Science and Research (MMIMSR), Mullana, Ambala, a 1200-bed tertiary care teaching hospital in northern Haryana, India. The study period extended from March 2025 to January 2026. Ethical clearance was obtained from the Institutional Ethics Committee (IEC) of MMIMSR prior to enrolment. Written informed consent was obtained from all patients or their legal guardians, in a language they understood.

Sample size

The sample size was estimated a priori using Cochran's formula for estimating a single proportion with specified precision [7], assuming an anticipated ICU mortality rate of 20% among patients with severe dengue based on prior regional literature [5,6], a two-sided alpha of 0.05 (Z = 1.96), and an absolute precision of ±8%. This yielded a minimum requirement of 96 patients. Enrolment was extended to 100 consecutive eligible patients to accommodate anticipated attrition; in the event, no patient was lost to follow-up.

Inclusion and exclusion criteria

Eligible patients were screened according to predefined inclusion and exclusion criteria, summarised in Table 1. A total of 100 consecutive patients fulfilling the WHO 2009 criteria for severe dengue and meeting all eligibility requirements were included in the final analysis.

**Table 1:** Inclusion and exclusion criteria NS1 - non-dtructural protein; ALT - alanine aminotransferase; AST - aspartate aminotransferase

Criteria type	Description
Inclusion criteria	Patients aged ≥18 years with confirmed dengue infection, evidenced by a positive dengue NS1 antigen and/or IgM antibody test, who fulfilled the WHO criteria for severe dengue. Severe dengue was defined as one or more of the following: severe plasma leakage leading to shock or fluid accumulation with respiratory distress; severe bleeding; severe organ impairment (AST or ALT >1,000 IU/L or ALT >10 times the upper limit of normal for age); impaired level of consciousness; involvement of the heart or other organs.
Exclusion criteria	Patients not fulfilling the WHO criteria for severe dengue; patients who did not provide informed consent for participation in the study.

Data collection and methodology

All baseline hemodynamic, biochemical, and severity-score parameters were recorded within six hours of ICU or emergency admission on a pre-designed structured proforma completed prospectively by the treating physician. Demographics, comorbidities, and warning signs (lethargy, abdominal pain, fluid accumulation, bleeding manifestations, hepatomegaly >2 cm, rising hematocrit with falling platelets) were documented at admission, along with complete blood count, liver and renal function tests, arterial blood gas with lactate, PT-INR, APTT, serum ferritin, GCS, and SOFA/APACHE II scores. Vital signs, GCS, and laboratory parameters were reassessed at 24-hour intervals until discharge or death; only admission values were analyzed against outcome.

Organ-specific complications (ARDS, MODS, AKI, hepatitis (aspartate aminotransferase or alanine aminotransferase >1000 IU/L or ALT > 10 times the upper limit of normal for age), encephalitis, myocarditis, disseminated intravascular coagulation (DIC), acute liver failure, seizures) and ICU interventions (vasopressor use, invasive/non-invasive ventilation) were recorded throughout the stay. Fluid resuscitation, vasopressor initiation, and ventilatory support followed the institutional MMIMSR critical care protocol for dengue shock, aligned with WHO 2009 and national guidelines; treatment decisions rested with the treating intensivist and were not otherwise standardized.

Patients were followed daily until ICU discharge or death, with outcome ascertained as survival to ICU discharge or in-hospital death; no patient was lost to follow-up, and complete data were available for all 100 enrolled patients (0% missing), obviating the need for imputation. Laboratory samples were processed at the institutional clinical laboratory, accredited by the National Accreditation Board for Testing and Calibration Laboratories (NABL), within two hours of collection.

Operational definitions

Impaired consciousness was defined as GCS < 15 on admission. Respiratory distress was defined per the American Thoracic Society consensus (subjective dyspnoea plus objective signs: tachypnoea, accessory muscle use, and intercostal retraction). ARDS was diagnosed using the 2012 Berlin criteria [8]. MODS was defined as concurrent dysfunction of two or more organ systems identified during the ICU admission. Encephalitis was defined as altered sensorium and/or seizures with supportive cerebrospinal fluid or neuroimaging findings suggestive of brain parenchymal inflammation. AKI was defined using the Kidney Disease: Improving Global Outcomes (KDIGO) criteria [9-11].

Statistical analysis

Data were analyzed using SPSS Statistics version 27.0 (IBM Inc., Armonk, NY, USA). Categorical variables are reported as frequencies and percentages; continuous variables as mean ± SD or median (interquartile range (IQR)). Between-group comparisons (survivors vs. non-survivors) for categorical variables used the chi-squared or Fisher exact test, as appropriate; continuous variables were compared using the independent-samples t-test or Mann-Whitney U test.

For binary categorical comparisons, mortality risk is additionally expressed using row percentages - the proportion of patients with a given clinical characteristic who died, versus the proportion without that characteristic who died - together with relative risk (RR) and 95% confidence intervals (CI). The Haldane-Anscombe continuity correction (addition of 0.5 to each cell of the 2×2 table) was applied where a zero-cell frequency precluded direct RR estimation; this is noted alongside the relevant estimates.

Effect sizes for chi-squared/Fisher-based associations were classified using the phi coefficient (φ) according to Cohen's conventions (φ <0.10 negligible, 0.10-0.29 small, 0.30-0.49 moderate, ≥0.50 large) [12]. Effect sizes for Mann-Whitney U comparisons were classified using the rank-biserial correlation (r), applying the equivalent thresholds described by Rosenthal (r <0.10 negligible, 0.10-0.29 small, 0.30-0.49 moderate, ≥0.50 large) [13]. ROC curve analysis was performed to assess the discriminative ability of clinical, laboratory, and severity-score parameters for ICU mortality; the area under the curve (AUC) is reported for each parameter. A p-value <0.05 was considered statistically significant throughout.

## Results

Study population and baseline characteristics

A total of 100 consecutive patients with severe dengue were enrolled in the study. The mean age was 47.20 ± 17.51 years (range: 18-84 years), and 58 patients (58.0%) were male. Comorbidities were present in 48 patients (48.0%), with diabetes mellitus (26/48, 54.2%) and hypertension (23/48, 47.9%) being the most common. No individual comorbidity was significantly associated with mortality. The overall ICU mortality rate was 21.0% (21/100 patients).

Fever was the most common presenting symptom, reported in 87 patients (87.0%), followed by shortness of breath in 30 (30.0%), vomiting in 27 (27.0%), generalised body ache in 21 (21.0%), altered sensorium in 15 (15.0%), loose stools in 14 (14.0%), and abdominal pain in 14 (14.0%) patients.

As presented in Table 2, lethargy was the most common WHO warning sign, occurring in 88 (88.0%) patients, followed by fluid accumulation in 69 (69.0%), abdominal pain in 58 (58.0%), respiratory distress in 52 (52.0%), bleeding manifestations in 40 (40.0%), and impaired consciousness in 29 (29.0%) patients. Among organ involvement, AKI was the most frequent complication, affecting 37 (37.0%) patients, followed by hepatitis in 22 (22.0%), MODS in 13 (13.0%), encephalitis in 12 (12.0%), and ARDS in 10 (10.0%) patients.

**Table 2:** Distribution of study subjects according to clinical warning signs and organ involvement (n=100) HCT - hematocrit; MODS - multiorgan dysfunction syndrome; ARDS - acute respiratory distress syndrome; DIC - disseminated intravascular coagulation

Parameter	Frequency, n (%)
Lethargy	88 (88.0)
Fluid accumulation	69 (69.0)
Abdominal pain	58 (58.0)
Respiratory distress	52 (52.0)
Bleeding manifestations	40 (40.0)
Impaired consciousness	29 (29.0)
HCT rise/platelet drop from baseline	17 (17.0)
Liver enlargement >2 cm	12 (12.0)
Organ involvement	
Acute kidney injury	37 (37.0)
Hepatitis	22 (22.0)
MODS	13 (13.0)
ARDS	10 (10.0)
Encephalitis	12 (12.0)
Myocarditis	2 (2.0)
DIC	1 (1.0)
Acute liver failure	1 (1.0)
Seizures	3 (3.0)

As presented in Table 3, thrombocytopenia was common in both survivors and non-survivors, with most patients having platelet counts between 20,000 and 50,000/mm³. Platelet count was not significantly associated with mortality (χ² = 1.47, df = 3, Cramer's V = 0.12, p = 0.678).

**Table 3:** Distribution of study subjects according to thrombocytopenia χ² = 1.47, df = 3, Cramer's V = 0.12, p = 0.678 (NS). Percentages are shown as column percentages (proportion within each outcome group χ² - chi-squared test statistic; df - degrees of freedom; NS - not significant

Platelet count (cells/mm³)	Non-survivors, n (%)	Survivors, n (%)	Total n (%)	p-value
< 20,000	3 (14.3)	17 (21.5)	20 (20.0)	0.678 (NS)
20,000 - 50,000	8 (38.1)	23 (29.1)	31 (31.0)	
50,000 - 100,000	3 (14.3)	17 (21.5)	20 (20.0)	
> 100,000 (normal/above)	7 (33.3)	22 (27.8)	29 (29.0)	

As presented in Table 4, higher pulse rate, respiratory rate, and body temperature were each significantly associated with an increased risk of ICU mortality (all p < 0.05), whereas higher systolic and diastolic blood pressure, GCS score, arterial pH, and PaO₂ were each significantly associated with a reduced risk of death. Total leucocyte count, serum creatinine, total bilirubin, and lactate were each significantly higher among patients who died. Alanine aminotransferase (ALT) and aspartate aminotransferase (AST) did not differ significantly between groups. Serum lactate, arterial pH, GCS, FiO₂, and total bilirubin showed the largest effect sizes (rank-biserial r ≥ 0.40), indicating a moderate-to-strong association with mortality.

**Table 4:** Association of hemodynamic and biochemical parameters with outcome * p < 0.05 NS - not significant; SD - standard deviation; IQR - interquartile range; U - Mann–Whitney U statistic; Z - standardized test statistic; r - rank-biserial effect size; BP - blood pressure; ALT - alanine aminotransferase; AST - aspartate aminotransferase; PaO₂ - partial pressure of oxygen; HCO₃⁻ - bicarbonate; FiO₂ - fraction of inspired oxygen

Parameter	Non-survivors, mean ± SD; median (IQR)	Survivors, mean ± SD; median (IQR)	U	Z	Effect size (r)	p-value
Pulse (per minute)	110.38 ± 16.34; 110.00 (99.00-125.00)	97.68 ± 15.62; 98.00 (88.00-110.00)	1187	3.025	0.303	0.001*
Systolic BP (mmHg)	85.47 ± 14.61; 84.00 (70.00-90.00)	107.39 ± 23.22; 110.00 (90.00-120.00)	594.5	−3.82	0.382	0.001*
Diastolic BP (mmHg)	56.21 ± 9.56; 60.00 (50.00-60.00)	64.35 ± 13.93; 70.00 (60.00-70.00)	701	−2.89	0.289	0.004*
Respiratory rate (per min)	30.19 ± 7.23; 28.00 (25.00-36.00)	25.87 ± 6.07; 26.00 (20.00-28.00)	1130.5	2.547	0.255	0.010*
Temperature (°F)	101.30 ± 1.43; 102.00 (100.0-102.0)	100.39 ± 1.33; 100.00 (100.0-102.0)	1109	2.365	0.237	0.014*
Glasgow Coma Scale	8.95 ± 3.05; 8.00 (7.50-11.50)	14.37 ± 1.94; 15.00 (15.00-15.00)	125	−5.962	0.596	0.001*
Platelet count (cells/mm³)	84,214 ± 74,365; 47,000 (35,000-125,500)	78,677 ± 73,574; 45,000 (24,000-109,000)	893.5	0.542	0.054	0.588 (NS)
Total leucocyte count (1000/mm³)	14.87 ± 8.06; 14.40 (7.54-19.09)	9.27 ± 8.11; 6.50 (4.21-11.69)	1221.5	3.317	0.332	0.001*
Creatinine (mg/dL)	2.27 ± 1.40; 1.92 (1.23-3.05)	1.59 ± 1.39; 1.02 (0.78-1.82)	1178.5	2.953	0.295	0.001*
Total bilirubin (mg/dL)	2.65 ± 2.26; 1.80 (1.01-3.30)	1.11 ± 1.31; 0.60 (0.40-1.40)	1313	4.092	0.409	0.001*
ALT (U/L)	815.80 ± 1753.00; 73.00 (22.50-455.00)	184.90 ± 363.76; 70.00 (38.00-169.00)	872	0.36	0.036	0.719 (NS)
AST (U/L)	1502.19 ± 3986.50; 104.00 (21.00-960.50)	265.24 ± 636.08; 101.00 (42.00-244.00)	874.5	0.381	0.038	0.703 (NS)
pH	7.125 ± 0.218; 7.149 (6.90-7.30)	7.344 ± 0.105; 7.400 (7.30-7.40)	291.5	−4.553	0.455	0.001*
PaO₂ (mmHg)	68.03 ± 14.44; 70.00 (59.50-76.50)	79.71 ± 10.67; 80.00 (75.00-88.00)	383.5	−3.774	0.377	0.001*
HCO₃⁻ (mmol/L)	15.45 ± 5.16; 14.00 (12.00-19.50)	17.20 ± 5.11; 16.50 (14.10-19.60)	650	−1.519	0.152	0.167 (NS)
FiO₂ (%)	81.29 ± 18.14; 82.00 (79.00-91.00)	92.87 ± 6.98; 94.00 (90.00-98.00)	476.5	−4.18	0.418	0.001*
Lactate (mmol/L)	5.61 ± 3.61; 5.40 (2.15-9.90)	1.99 ± 2.84; 1.30 (0.70-2.50)	1384.5	4.697	0.47	0.001*

As presented in Table 5, liver enlargement >2 cm was the only WHO warning sign significantly associated with mortality: 6 of 12 patients (50.0%) with liver enlargement died, compared with a substantially lower proportion among those without it (RR 2.93, 95% CI 1.41-6.09; p = 0.024). Lethargy, fluid accumulation, abdominal pain, bleeding manifestations, and HCT rise/platelet drop showed no statistically significant association with outcome

**Table 5:** WHO warning signs and their association with mortality * p < 0.05; df = 1 for all rows; † - Haldane-Anscombe continuity correction (+0.5/cell) applied for a zero cell NS - not significant; χ² - chi-squared (Fisher exact used where expected cell count <5); φ - phi coefficient; HCT - hematocrit; RR - relative risk; CI - confidence interval

Warning sign	Non-survivors, n (row %)	Survivors, n (row %)	Total n	χ²	Effect Size (φ)	p-value	RR (95% CI)
Lethargy	21 (23.9)	67 (76.1)	88	3.625	0.19	0.127 (NS)	6.28† (0.40-97.55)
Fluid accumulation	18 (26.1)	51 (73.9)	69	3.472	0.186	0.062 (NS)	2.70 (0.86-8.48)
Abdominal pain	14 (24.1)	44 (75.9)	58	0.82	0.091	0.360 (NS)	1.45 (0.64-3.27)
Bleeding manifestations	8 (20.0)	32 (80.0)	40	0.04	0.02	0.841 (NS)	0.92 (0.42-2.02)
HCT rise/platelet drop	4 (23.5)	13 (76.5)	17	0.079	0.028	1.000 (NS)	1.15 (0.44-2.99)
Liver enlargement >2 cm	6 (50.0)	6 (50.0)	12	6.913	0.263	0.024*	2.93 (1.41-6.09)

As presented in Table 6, impaired consciousness showed the strongest association with mortality: 21 of 29 patients (72.4%) with impaired consciousness died, compared with 0 of 71 (0%) without it (RR 103.20, 95% CI 6.46-1649.18; p < 0.001). All 10 patients who developed ARDS died (RR 7.55, 95% CI 4.33-13.16; p < 0.001), and 10 of 13 patients (76.9%) with MODS died (RR 6.08, 95% CI 3.25-11.39; p = 0.002). Shock (RR 9.79, 95% CI 3.09-31.03), respiratory distress (RR 8.77, 95% CI 2.16-35.68), and encephalitis (RR 4.51, 95% CI 2.38-8.57) were also significantly associated with mortality (all p < 0.001). Acute kidney injury, hepatitis, myocarditis, seizures, and DIC showed no significant association with outcome.

**Table 6:** Association of organ involvement of study subjects with outcome (n=100) * p < 0.05 (statistically significant); † = Haldane-Anscombe correction applied for a zero cell; the very wide CIs for impaired consciousness and ARDS reflect small-sample "perfect separation" and warrant cautious interpretation. NS - not significant; χ² - chi-squared test statistic (df = 1); φ - phi coefficient (Cohen thresholds [12]); RR - relative risk; CI - confidence interval; MODS - multiorgan dysfunction syndrome; ARDS - acute respiratory distress syndrome; DIC - disseminated intravascular coagulation

Organ complication / warning sign	Non-survivors, n (row %)	Survivors, n (row %)	Total n	χ²	Effect size (φ)	p-value	RR (95% CI)
Impaired consciousness	21 (72.4)	8 (27.6)	29	65.081	0.807	<0.001*	103.20† (6.46–1649.18)
ARDS	10 (100.0)	0 (0.0)	10	41.799	0.647	<0.001*	7.55† (4.33–13.16)
MODS	10 (76.9)	3 (23.1)	13	28.168	0.531	0.002*	6.08 (3.25–11.39)
Shock	18 (47.4)	20 (52.6)	38	25.687	0.507	0.001*	9.79 (3.09–31.03)
Respiratory distress	19 (36.5)	33 (63.5)	52	15.766	0.397	<0.001*	8.77 (2.16–35.68)
Acute kidney injury	11 (29.7)	26 (70.3)	37	2.698	0.164	0.100 (NS)	1.87 (0.88–3.98)
Encephalitis	8 (66.7)	4 (33.3)	12	17.142	0.414	<0.001*	4.51 (2.38–8.57)
Hepatitis	7 (31.8)	15 (68.2)	22	1.99	0.141	0.227 (NS)	1.77 (0.82–3.84)
Myocarditis	1 (50.0)	1 (50.0)	2	1.035	0.102	1.000 (NS)	2.45 (0.58–10.34)
Seizures	1 (33.3)	2 (66.7)	3	0.284	0.053	1.000 (NS)	1.62 (0.31–8.40)
DIC	0 (0.0)	1 (100.0)	1	0.269	0.052	1.000 (NS)	1.16† (0.10–13.20)

As presented in Table 7, vasopressor requirement was associated with a markedly increased risk of death: 18 of 37 patients (48.6%) who required vasopressors died (RR 10.22, 95% CI 3.23-32.36; p < 0.001). Requirement of ventilatory support carried the highest risk in the cohort, with 19 of 26 patients (73.1%) who received it dying (RR 27.04, 95% CI 6.76-108.21; p < 0.001). Within this group, invasive mechanical ventilation was itself significantly associated with death (10 of 11 patients ventilated invasively died, versus 11 of 89 not invasively ventilated; RR 7.36, 95% CI 4.10-13.19; χ² = 36.410, φ = 0.603; p < 0.001), whereas non-invasive ventilation, used only in patients with less severe respiratory compromise, showed no significant association with outcome (0 of 4 patients receiving non-invasive ventilation died; RR 0.45, 95% CI 0.03-6.42; χ² = 1.108, φ = 0.105; p = 0.577, NS).

**Table 7:** Supportive care interventions and mortality p < 0.05; ° Fisher exact test used (expected cell count <5); † - Haldane-Anscombe correction applied for the zero cell in non-invasive ventilation. NS - not significant; χ² - chi-squared; φ - phi coefficient (effect size); RR - relative risk; CI - confidence interval;

Intervention	Non-survivors, n ( %)	Survivors, n ( %)	Total n	χ²	φ	p-value	RR (95% CI)
Vasopressor requirement	18 (48.6)	19 (51.4)	37	27.062	0.52	<0.001*	10.22 (3.23-32.36)
Any ventilatory support	19 (73.1)	7 (26.9)	26	57.436	0.758	<0.001*	27.04 (6.76-108.21)
Invasive mechanical ventilation°	10 (90.9)	1 (9.1)	11	36.41	0.603	<0.001*	7.36 (4.10-13.19)
Non-invasive ventilation°	0 (0.0)	4 (100.0)	4	1.108	0.105	0.577 (NS)	0.45† (0.03-6.42)

As presented in Table 8, non-survivors had significantly higher SOFA and APACHE II scores, INR, prothrombin time, and serum ferritin than survivors (all p < 0.05). SOFA score (r = 0.615) and APACHE II score (r = 0.611) demonstrated the largest effect sizes, followed by INR (r = 0.452), reinforcing their value as the factors most strongly associated with ICU mortality in this cohort. PT showed a moderate association (r = 0.344) and ferritin a small-to-moderate association (r = 0.291) with outcome, while APTT did not differ significantly between groups (p = 0.061).

**Table 8:** Association of severity scores and coagulation parameters with outcome * p < 0.05; Effect size r is the rank-biserial correlation from the Mann–Whitney U test, classified per Rosenthal (1991): <0.10 negligible, 0.10-0.29 small, 0.30-0.49 moderate, ≥0.50 large. NS - not significant; SOFA - Sequential Organ Failure Assessment; APACHE II - Acute Physiology and Chronic Health Evaluation II; INR - international normalized ratio; PT - prothrombin time; APTT - activated partial thromboplastin time

Parameter	Non-survivors, mean ± SD; median (IQR)	Survivors, mean ± SD; median (IQR)	Test statistic (U)	r	p-value
SOFA score	12.90 ± 3.754; 13.00 (11.00-16.00)	5.00 ± 2.67; 5.00 (3.00-6.00)	1556.5	0.615	0.001*
APACHE II score	26.43 ± 8.109; 25.00 (21.50-33.00)	9.80 ± 6.94; 8.00 (4.00-15.00)	1551.5	0.611	0.001*
INR	1.71 ± 0.71; 1.50 (1.21-2.30)	1.10 ± 0.34; 1.06 (0.94-1.20)	1364	0.452	0.001*
PT (seconds)	18.24 ± 7.56; 15.60 (13.40-21.25)	13.44 ± 5.29; 12.00 (10.80-15.00)	1236	0.344	0.001*
APTT (seconds)	43.20 ± 16.14; 40.00 (31.50-53.60)	36.73 ± 14.55; 34.00 (28.00-40.00)	1050.5	0.187	0.061 (NS)
Ferritin (ng/mL)	2565.62 ± 3601.69; 1800.00 (1540.00-2095.00)	1544.42 ± 2249.90; 1085.00 (540.00-2000.00)	1173.5	0.291	0.004*

On ROC curve analysis of baseline parameters against outcome, SOFA score demonstrated the highest discriminative ability (AUC = 0.938, 95% CI 0.869-1.000; sensitivity 85.71%, specificity 96.20% at a cut-off of ≥10), followed closely by APACHE II score (AUC = 0.935, 95% CI 0.882-0.988; sensitivity 85.71%, specificity 92.41% at a cut-off of ≥19). Both scores showed excellent discrimination by conventional AUC benchmarks (AUC ≥ 0.9). Among individual parameters, lactate (AUC = 0.835, 95% CI 0.726-0.944) and arterial pH (AUC = 0.824, 95% CI 0.718-0.930) showed the next-highest discrimination, followed by total bilirubin (AUC = 0.791, 95% CI 0.687-0.896) and systolic blood pressure (AUC = 0.782, 95% CI 0.683-0.882). Pulse rate, respiratory rate, temperature, and diastolic blood pressure showed fair discrimination (AUC 0.67-0.72). Packed cell volume, AST, and ALT yielded AUC values close to 0.5, indicating little to no discriminative value at the cut-offs tested; platelet count similarly showed weak discrimination (AUC = 0.539, 95% CI 0.397-0.680).

## Discussion

This prospective observational cohort study from a tertiary-care ICU in North India provides important insights into the factors associated with mortality in severe dengue and supports closer attention to indicators of organ dysfunction, tissue hypoperfusion, and systemic decompensation along with traditional hematological markers. Our findings suggest that adverse outcomes in severe dengue are associated predominantly with progressive multi-organ involvement and hemodynamic instability rather than with thrombocytopenia or hepatic enzyme derangements alone.

The clinical profile of the cohort was characterized by a high prevalence of lethargy, fluid accumulation, respiratory distress, and bleeding manifestations. Among organ complications, AKI was the most frequent, followed by hepatitis, MODS, encephalitis, and ARDS (Table 2). These findings highlight the predominance of plasma leakage and organ dysfunction in severe dengue, underscoring its multisystem involvement.

An important finding of this study was the lack of association between hematological parameters and mortality. As shown in Table 3, platelet count - the parameter most frequently monitored and acted upon in dengue management across India -was not associated with survival status in ICU-admitted severe dengue (p = 0.678). This is consistent with the 2021 systematic review by Htun et al., which found that thrombocytopenia and AST elevation, while associated with severe dengue classification, were not independently predictive of mortality when organ dysfunction variables were included [4]. Our study adds the specific ICU context: among patients who have already met WHO severe-dengue criteria and been admitted to ICU, the hematological trajectory is not strongly associated with survival. As shown in Table 5, bleeding manifestations similarly showed no association with mortality (p = 0.841), reinforcing that hemorrhagic manifestations of dengue are not the primary mechanism by which most ICU patients in this cohort died.

A striking observation of the present study was the markedly different physiological profile of survivors and non-survivors. As shown in Table 4, several hemodynamic and biochemical parameters were significantly associated with mortality in patients with severe dengue. These differences collectively describe a pattern of circulatory failure, metabolic acidosis, tissue hypoperfusion, and progressive organ dysfunction preceding death. ALT and AST levels did not differ significantly between groups, suggesting that conventional hepatic markers have limited discriminatory value in critically ill dengue patients compared with markers of organ dysfunction and physiological derangement.

The most compelling finding of the present study was the strong association between specific organ complications and mortality. As shown in Table 6, 21 of the 29 patients with impaired consciousness died (72.4%; χ² = 65.081, φ = 0.807, p < 0.001), making it the clinical feature most strongly associated with death in this cohort. The mean GCS of 8.95 in non-survivors - representing moderate-to-severe neurological impairment - contrasts sharply with a mean of 14.37 in survivors. The large effect size further highlights the potential clinical importance of routine neurological assessment during initial evaluation. The mechanisms underlying neurological involvement in severe dengue are multifactorial: cerebral hypoperfusion from dengue shock syndrome, metabolic encephalopathy from acidosis and hyperlactataemia, direct neurotropism of the dengue virus, and immune-mediated cerebral inflammation may each contribute to reduced consciousness [14]. These findings align with Jain et al. from North India, who developed a mortality prediction score in which altered sensorium carried the highest regression coefficient [15], and with Chen et al. from Taiwan, who similarly identified lower GCS as a significant multivariable predictor of mortality in critically ill dengue patients [16].

The absolute exclusivity of ARDS to non-survivors in our cohort - a 0% survivor ARDS rate - is a finding not previously reported in a consecutive North Indian ICU series and provides strong discriminatory evidence, although the wide confidence interval around this estimate (Table 6) reflects the small number of ARDS cases and should be interpreted cautiously. Dengue-associated ARDS results from diffuse alveolar damage driven by plasma leakage into the pulmonary interstitium, cytokine-mediated endothelial injury, and secondary pneumonia [17]. The Réunion Island ICU series by Vidal et al. reported a 38% mortality in ICU-admitted severe dengue patients and identified respiratory rate as an independent predictor of death on multivariable analysis [6]. Our finding extends this observation by demonstrating that when the ARDS threshold was met per the 2012 Berlin Criteria, the outcome was uniformly fatal in our cohort [8].

MODS represents a convergent pathway by which severe dengue is associated with death. The pattern of impaired consciousness, respiratory failure, and multi-system organ dysfunction tracks the pathophysiological cascade from dengue shock (tissue hypoperfusion leading to lactic acidosis, followed by neurological and respiratory decompensation) to death. This is broadly consistent with the Réunion Island ICU cohort described above, in which organ dysfunction parameters accounted for a substantial portion of the mortality signal [6]. Padyana et al. from South India similarly reported that ARDS, AKI, and shock were the clinical syndromes most strongly associated with mortality in dengue ICU patients, while hepatic involvement was near-universal and not discriminatory [18]; Shashtri et al., in a North Indian ICU cohort, reported broadly comparable findings [19].

ROC analysis showed that SOFA and APACHE II scores had the highest discriminative performance for ICU mortality in severe dengue, consistent with mortality-prediction models described in other severe dengue cohorts [20]. Among laboratory parameters, total bilirubin, arterial pH, lactate, and serum creatinine demonstrated fair discriminative performance, reflecting the importance of multiorgan dysfunction, whereas platelet count, hematocrit, AST, and ALT showed poor discriminatory ability. These findings support the value of severity scores and organ-dysfunction markers for early prognostic assessment in severe dengue, while underscoring that thrombocytopenia alone has little discriminatory value for mortality.

The clinical implications of these findings should be interpreted cautiously given the single-center, observational design and the absence of multivariable adjustment. With this caveat, the pattern of associations observed suggests that ICU monitoring protocols for severe dengue may benefit from: (1) serial GCS assessment, with a declining score prompting reassessment of cerebral perfusion and metabolic status; (2) daily PaO₂/FiO₂ calculation, with early identification of ARDS prompting consideration of lung-protective ventilation strategies; and (3) daily SOFA scoring, with a rising trajectory prompting escalation of organ support. The absence of a mortality association with platelet count in this cohort suggests that routine platelet monitoring frequency and transfusion thresholds could be revisited, though this should first be confirmed in larger, multicentre studies employing multivariable analysis before any change in current practice is adopted.

Limitations

This study has several limitations. It was conducted at a single tertiary-care center with a sample of 100 patients, drawn from a referral ICU with likely selection bias toward the most severe presentations, which may limit generalisability to lower-acuity settings; dengue serotype data were not available. Although the required sample size was estimated a priori, the study was not powered to support multivariable modeling: with only 21 deaths, the widely used threshold of ten events per candidate predictor variable would have permitted at most two covariates in an adjusted model, which was judged insufficient to represent the range of organ-dysfunction variables identified on univariable testing. Multivariable logistic regression, with adjusted odds ratios and confidence intervals, has therefore been deferred to a planned, adequately powered multicentre extension of this work.

Consequently, the associations reported here - including the finding that impaired consciousness, ARDS, and MODS were each linked to mortality - should be interpreted as unadjusted factors associated with outcome rather than independent or causal predictors. The wide confidence intervals around several relative risks (impaired consciousness and ARDS, both of which showed zero-cell counts in their respective 2×2 tables) reflect this sparse-data instability, and the apparent complete separation between outcome groups for these variables is, in part, a small-sample phenomenon rather than an absolute clinical threshold that can be assumed to generalize. The observational cohort design further precludes causal inference. Validation of these findings in larger, multicentre cohorts with multivariable analysis is required before they can inform changes in clinical monitoring or management practice.

## Conclusions

This prospective observational cohort study from a North Indian tertiary-care ICU shows that impaired consciousness, ARDS, and MODS constitute a triad of factors strongly and consistently associated with mortality in severe dengue, whereas thrombocytopenia and bleeding manifestations showed no such association. Among the classical WHO warning signs, only liver enlargement was linked to outcome. These findings support increased attention to neurological status and organ-dysfunction assessment alongside conventional hematological surveillance in the ICU management of severe dengue, and provide a basis for these prognostic markers to be validated in larger, multicentre studies incorporating multivariable analysis.

## References

[1] (2026). Dengue and severe dengue. https://www.who.int/news-room/fact-sheets/detail/dengue-and-severe-dengue.

[2] (2026). Dengue: guidelines for diagnosis, treatment, prevention and control. https://www.who.int/publications/i/item/9789241547871.

[3] Shepard DS, Undurraga EA, Halasa YA (2013). Economic and disease burden of dengue in Southeast Asia. PLoS Negl Trop Dis.

[4] Htun TP, Xiong Z, Pang J (2021). Clinical signs and symptoms associated with WHO severe dengue classification: a systematic review and meta-analysis. Emerg Microbes Infect.

[5] Nivetha S, Kumar A, Eshwari K, Shetty A, Adarsha GK, Saravu K (2025). Clinico-epidemiological determinants of severe dengue in an endemic district of coastal Karnataka. Infect Dis (Lond).

[6] Vidal C, Soupin-Coulin Q, Doucet W (2025). Severe dengue in adults admitted to intensive care units on réunion island: clinical spectrum, outcomes, and predictors of mortality. Crit Care.

[7] Cochran WG (1977). Sampling Techniques.

[8] Ranieri VM, Rubenfeld GD, Thompson BT (2012). Acute respiratory distress syndrome: the Berlin definition. JAMA.

[9] (2012). KDIGO clinical practice guideline for acute kidney injury. Kidney Int Suppl.

[10] Khwaja A (2012). KDIGO clinical practice guidelines for acute kidney injury. Nephron Clin Pract.

[11] Marshall JC (2022). Multiple organ dysfunction syndrome. https://accessmedicine.mhmedical.com/book.aspx?bookID=3095.

[12] Cohen J (1988). Statistical Power Analysis for the Behavioral Sciences. Lawrence Erlbaum Associates.

[13] Rosenthal R (1991). Meta-analytic Procedures for Social Research.

[14] Carod-Artal FJ, Wichmann O, Farrar J, Gascón J (2013). Neurological complications of dengue virus infection. Lancet Neurol.

[15] Jain S, Mittal A, Sharma SK (2017). Predictors of dengue-related mortality and disease severity in a tertiary care center in North India. Open Forum Infect Dis.

[16] Chen CM, Chan KS, Yu WL, Cheng KC, Chao HC, Yeh CY, Lai CC (2016). The outcomes of patients with severe dengue admitted to intensive care units. Medicine (Baltimore).

[17] Keshav LB, K A, Malhotra K, Shetty S (2024). Lung manifestation of dengue fever: a retrospective study. Cureus.

[18] Padyana M, Karanth S, Vaidya S, Gopaldas JA (2019). Clinical profile and outcome of dengue fever in multidisciplinary intensive care unit of a tertiary level hospital in India. Indian J Crit Care Med.

[19] Shastri PS, Gupta P, Kumar R (2020). A prospective 3 year study of clinical spectrum and outcome of dengue fever in ICU from a tertiary care hospital in North India. Indian J Anaesth.

[20] Md-Sani SS, Md-Noor J, Han WH (2018). Prediction of mortality in severe dengue cases. BMC Infect Dis.

